# Corrigendum: Rapid generation of hypomorphic mutations

**DOI:** 10.1038/ncomms14705

**Published:** 2017-02-16

**Authors:** Laura L. Arthur, Joyce J. Chung, Preetam Janakirama, Kathryn M. Keefer, Igor Kolotilin, Slavica Pavlovic-Djuranovic, Douglas L. Chalker, Vojislava Grbic, Rachel Green, Rima Menassa, Heather L. True, James B. Skeath, Sergej Djuranovic

Nature Communications 8: Article number: 14112; DOI: 10.1038/ncomms14112 (2017); Published: 07 15 2015; Updated: 02 16 2017

The original version of this Article contained a typographical error in the spelling of the Author Preetam Janakirama, which was incorrectly given as Preetam Jankirama. This has now been corrected in both the PDF and HTML versions of the Article.

