# Supplementary material for: Rapid generation of hypomorphic mutations
Source: Nat Commun. 2017 Jan 20;8:14112. doi: 10.1038/ncomms14112 (PMC5263891; doi:10.1038/ncomms14112)

Supplementary Figure 1.

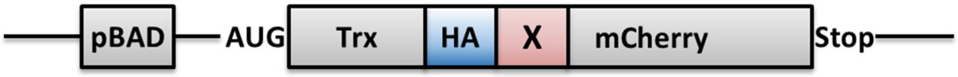

X = 6-12xLys$_{AAG}$ or 3-12Lys$_{AAA}$

WT **(TRX-HA) – Ala – Val – (mCherry)**
- GCA - GTG -

(AAG)$_n$ **(TRX-HA) – Ala – (Lys)$_{6\text{-}12}$ – Val – (mCherry)**
- GCA - (AAG)$_{6\text{-}12}$ - GTG -

(AAA)$_n$ **(TRX-HA) – Ala – (Lys)$_{6\text{-}12}$ – Val – (mCherry)**
- GCA - (AAA)$_{6\text{-}12}$ - GTG -

Diagram of mCherry expression construct used in *E. coli*. Position of the inducible arabinose promoter (pBAD), Thioredoxin (Trx), double HA-tag (HA), insertion sequence (X) and fluorescent reporter (mCherry) are indicated. Examples of WT, Lys$_{AAG}$ and Lys$_{AAA}$ insertions and the resulting protein and DNA sequences of reporter constructs are shown.

Supplementary Figure 2.

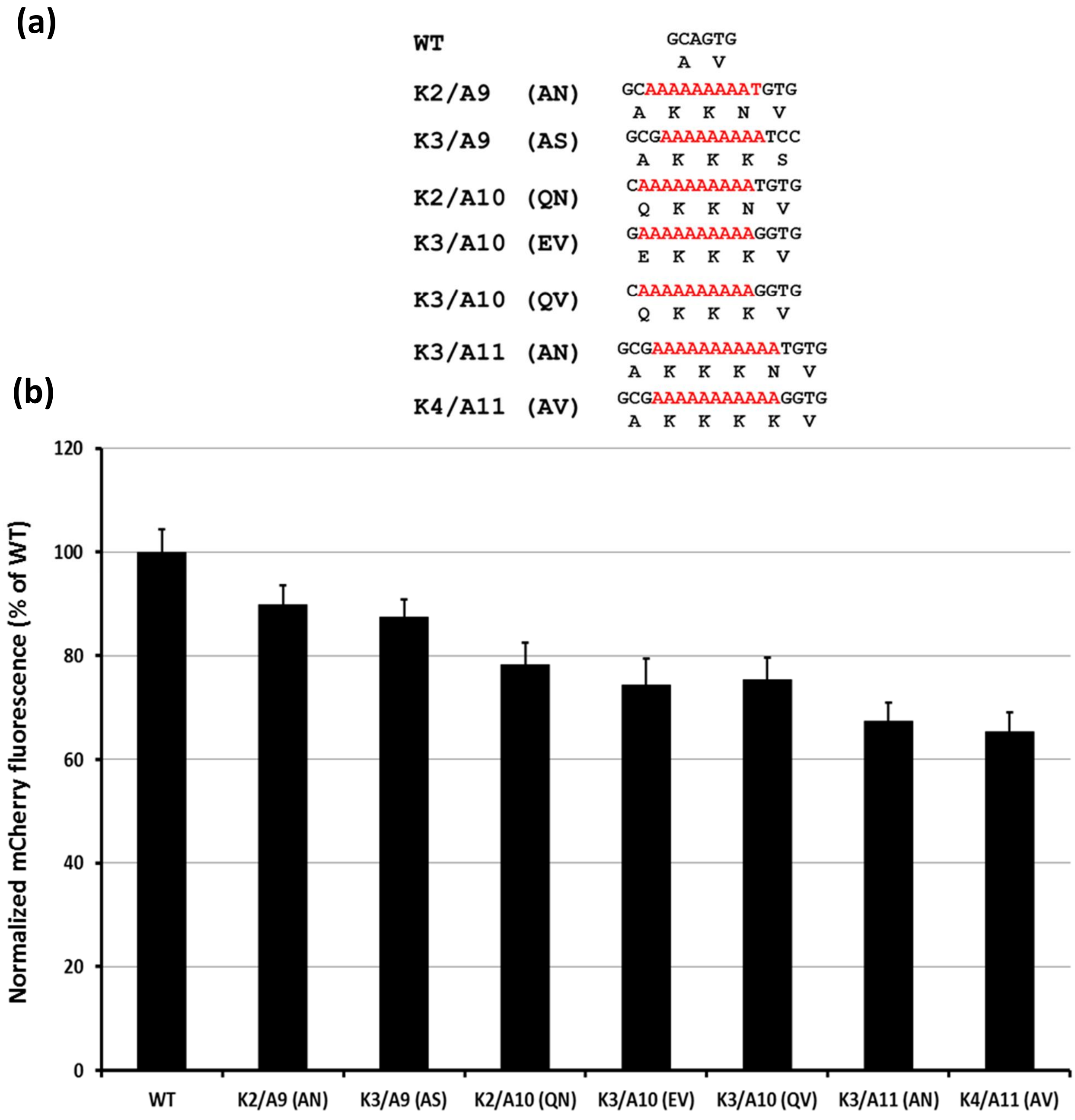

Supplementary Figure 2.

(c)

33As	-GTACCGGATTATGCGAAAAAAAAAAAAAAAAAAAAAAAAAAAAAAAAAAGTGAGCAAGG-	$(Lys)_{11}$
15A(CTG)15A	-GTACCGGATTATGCGAAAAAAAAAAAAAAAACTGAAAAAAAAAAAAAAAGTGAGCAAGG-	$(Lys)_{5}(Arg)(Lys)_{5}$
15A(TAC)15A	-GTACCGGATTATGCGAAAAAAAAAAAAAAAATACAAAAAAAAAAAAAAAGTGAGCAAGG-	$(Lys)_{5}(Tyr)(Lys)_{5}$
15A(CCC)15A	-GTACCGGATTATGCGAAAAAAAAAAAAAAAACCCAAAAAAAAAAAAAAAGTGAGCAAGG-	$(Lys)_{5}(Pro)(Lys)_{5}$
30As	-GTACCGGATTATGCGAAAAAAAAAAAAAAAA---AAAAAAAAAAAAAAAGTGAGCAAGG-	$(Lys)_{10}$

(d)

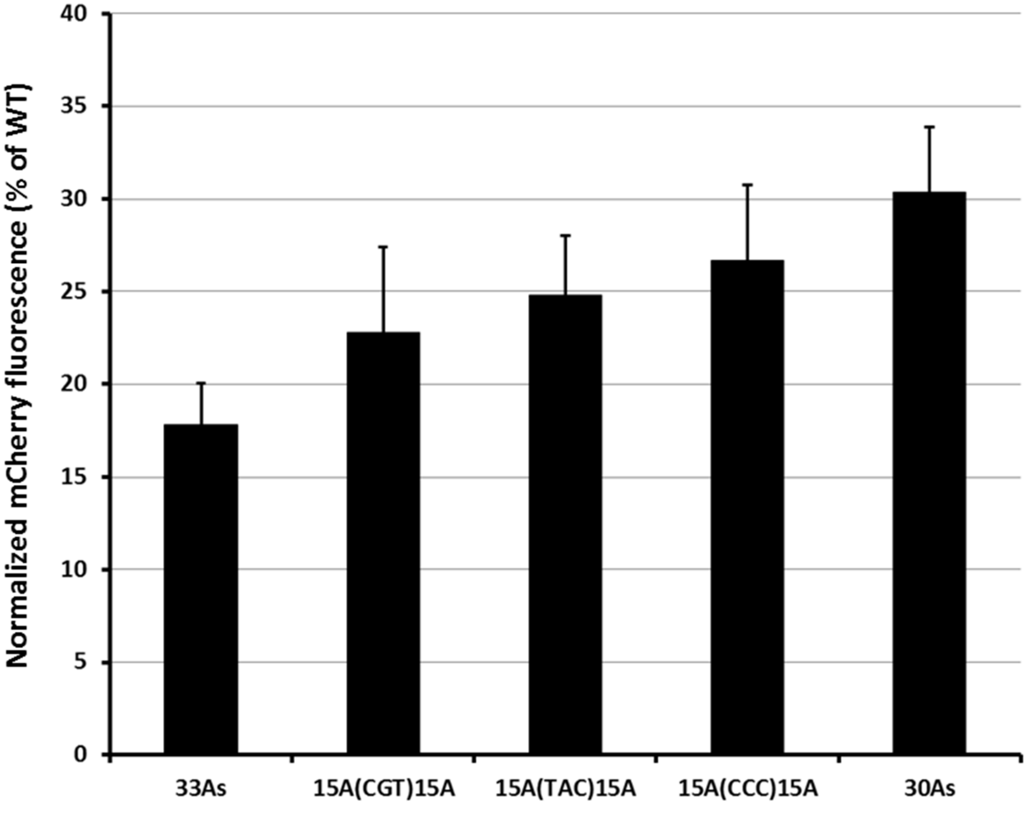

Quantification of mCherry fluorescence with modified polyA track sequences. PolyA tracks designed with flanking XAA and AAY codons, where X and Y denote C/G or T/C/G nucleotides respectively, were inserted into the mCherry reporter. The number of lysine residues (K) and adenosine residues (A) are noted as well as the two amino acids flanking lysine. (a). Normalized mCherry fluorescence intensity. Error bars represent #### (b). PolyA tracks with non-lysine codons interrupting the consecutive AAA codons were inserted into the mCherry reporter. The number of adenosine residues and interrupting codon and the protein sequences are indicated (c). Normalized mCherry fluorescence intensity. Error bars represent standard deviation from three different colonies (b and d).

Supplementary Figure 3.

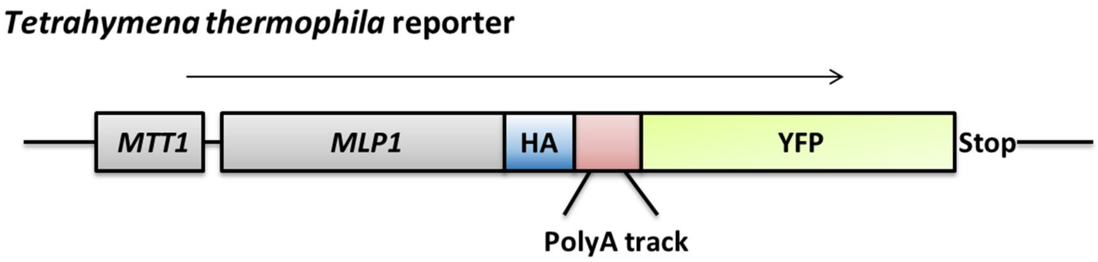

Diagram of mCherry expression construct used in *T. thermophila*. Position of the inducible metallothionein promoter (MTT1), Macronucleus-Localized Protein 1 (MLP1), double HA-tag (HA) and fluorescent reporter (eYFP) are indicated. Red box designates the position of in frame inserted polyA tracks and 12 Lys_{AAG} sequences. WT construct contains no insertions at this position.

Supplementary Figure 4.

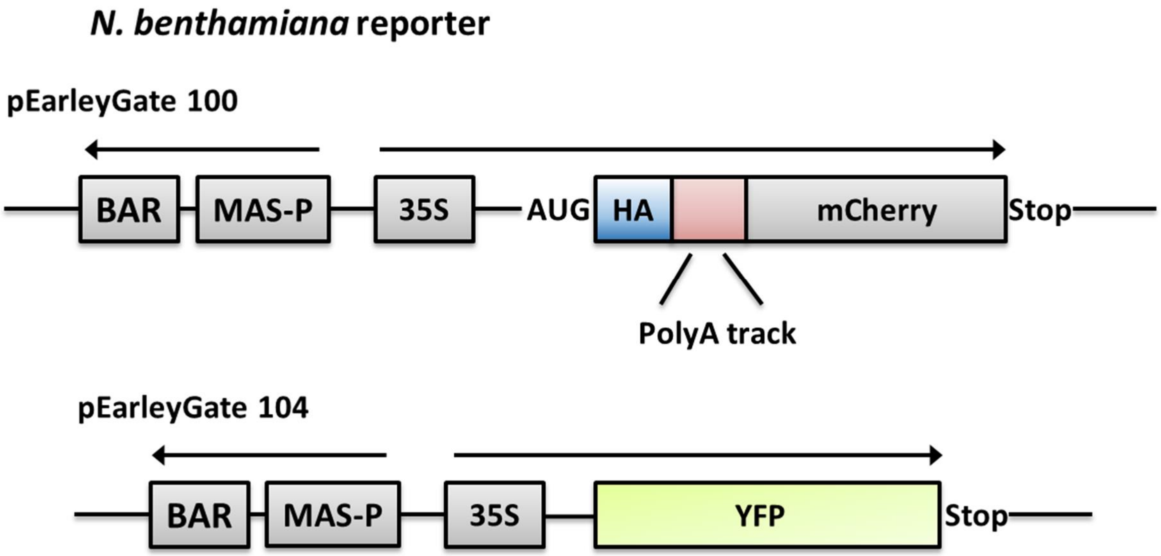

Diagram of mCherry and YFP expression constructs used in *N. benthamiana*. Position of the mannopine synthase promoter (MAS-P), the cauliflower mosaic virus 35S promoter and its upstream enhancer(35S), phosphinotricin acetyl transferase (BAR, herbicide resistance gene for selection of transgenic plants), double HA-tag (HA) and fluorescent reporters, mCherry and YFP, are indicated. Red box designates the position of in frame inserted polyA tracks and 12 Lys_{AAG} sequences. WT construct contains no insertions at this position.

Supplementary Figure 5.

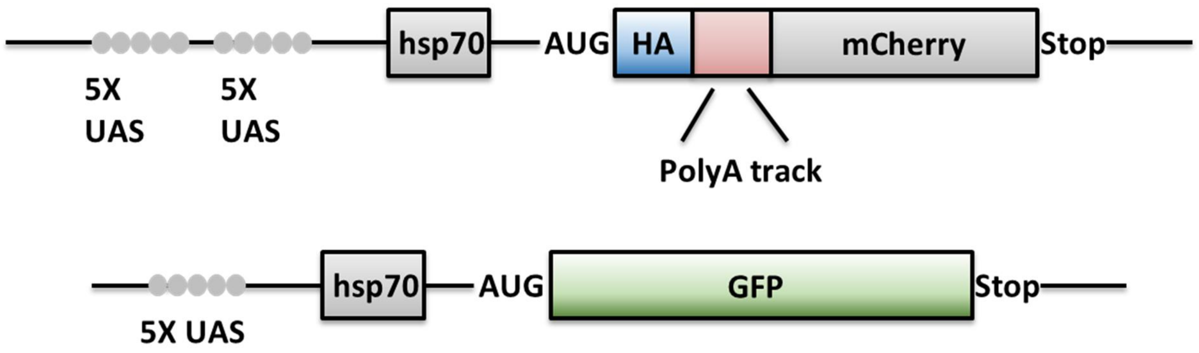

Diagram of mCherry and GFP expression constructs used in *D. melanogaster*. Position of the heat shock protein 70 promoter (hsp70), upstream activating sequences (UAS, GAL4 DNA binding sequence), double HA-tag (HA) and fluorescent reporters, mCherry and GFP, are indicated. Red box designates the position of in frame inserted polyA tracks and 12 Lys_{AAG} sequences. WT construct contains no insertions at this position. Tub-GAL4 driver line used for the expression of mCherry and GFP was derived from BSC42734.

Supplementary Figure 6.

(a)

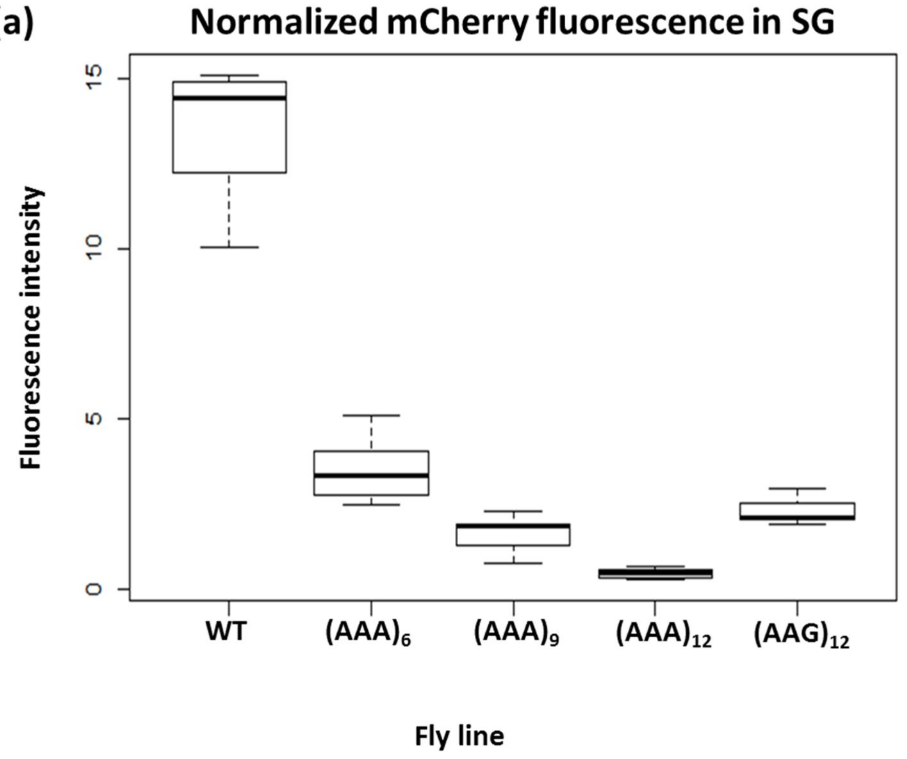

(b)

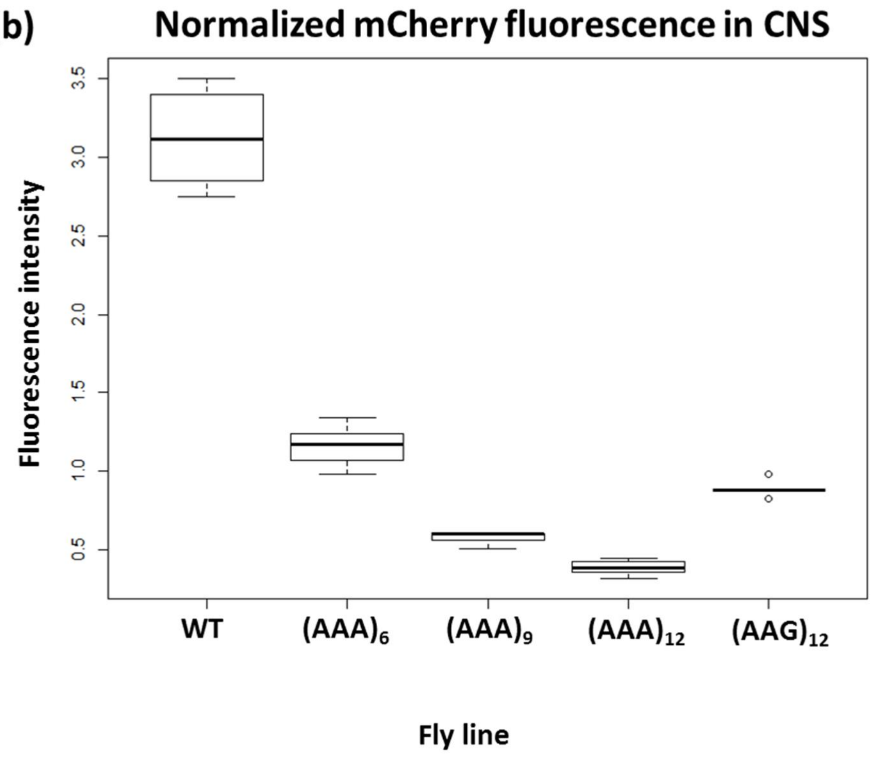

Supplementary Figure 6.

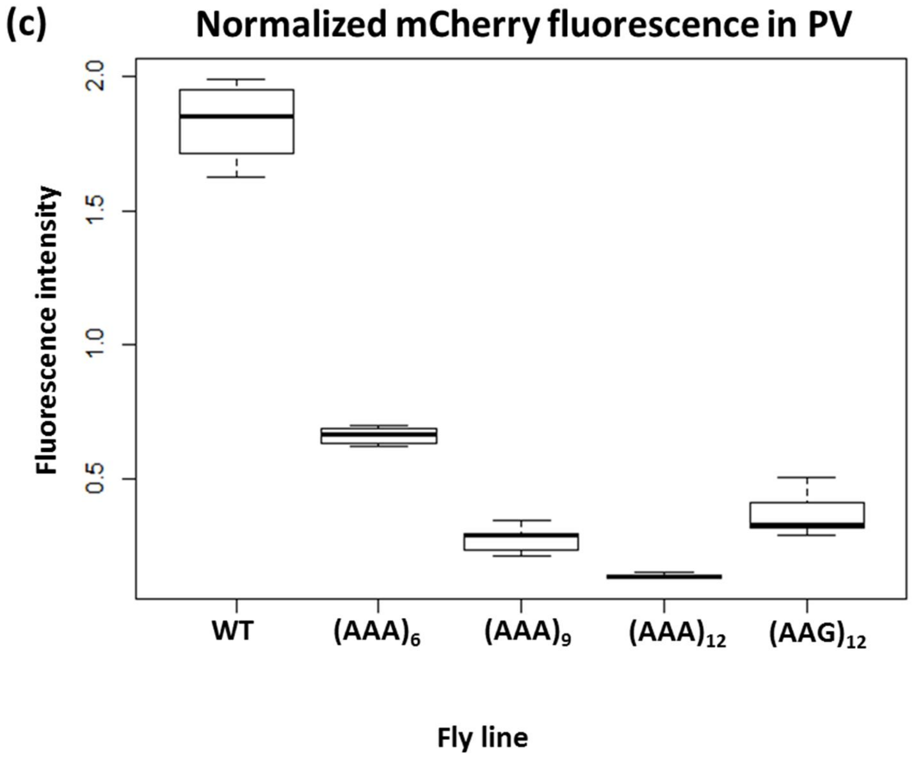

Quantification of mCherry fluorescence in *D. melanogaster* Salivary Glands (SG), Central Nervous System (CNS), and Proventriculus (PV). Normalized mCherry fluorescence intensity of WT, 12 Lys_{AAG} and 6-12 Lys_{AAA} in *D. melanogaster* SG (a), CNS (b) and PV (c). GFP fluorescence was excited by a 488 nm laser and mCherry by a 561nm laser. All microscopy parameters were constant between tissues, except master gain which was set as follows: SG – 488nm laser master gain was 509, 561 nm laser was 560, CNS - 488nm laser master gain was 625, 561 nm laser was 720, and PV - 488nm laser master gain was 618, 561 nm laser was 616. Fluorescence intensity was measured as an average intensity from each tissue image (Zen 9 software) and plotted as a ratio of mCherry to GFP intensity. Box plots indicate median intensity ratio per construct (n≥5).

Supplementary Figure 7.

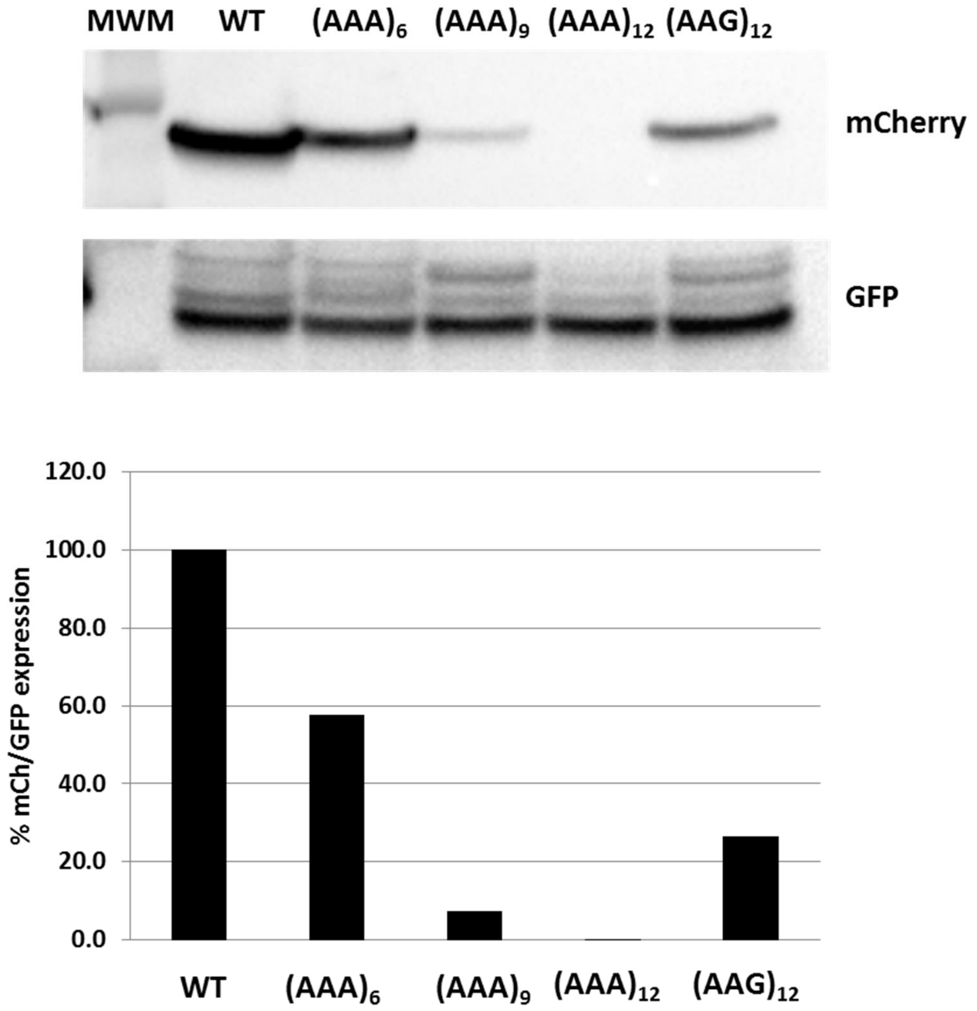

Western blot analysis and quantification of mCherry protein from third instar *D. melanogaster* larvae. Five third instar fruit fly larvae expressing either WT, 12 Lys_{AAG} or 6-12Lys_{AAA} constructs were frozen, homogenized, sonicated and lysed in SDS sample buffer. Equal amounts of lysate were analyzed by SDS-PAGE followed by western blot transfer. mCherry protein was detected using HA-tag antibody (Santa Cruz Biotechlogy Inc) and relative amounts were calculated based on the GFP expression control. GFP protein was detected using GFP specific antibody (Clontech). Relative amounts of mCherry expression are shown as percentage of WT-mCherry expression.

Supplementary Figure 8.

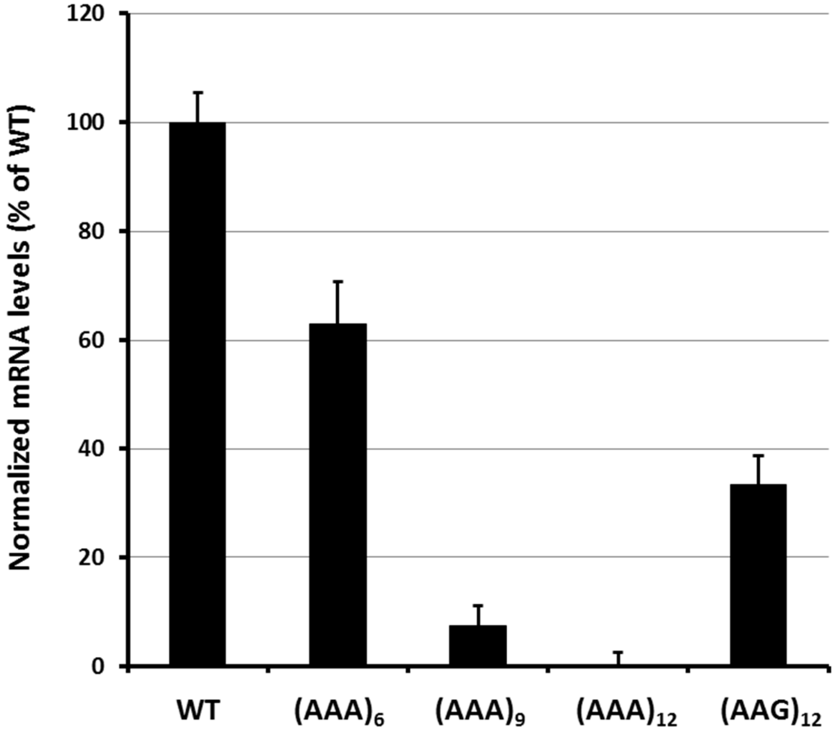

mCherry mRNA abundance in whole third instar D. melanogaster larvae measured by RT-qPCR. Five third instar fruit fly larvae expressing either WT, 12 Lys_{AAG} or 6-12Lys_{AAA} constructs were frozen, homogenized, and lysed in RiboZol (Ambion). mCherry RNA abundance was measured by RT-qPCR. Relative amounts of mCherry mRNA were normalized to levels of Elongation Factor 1 alpha-100 (EF1) and shown as percentage of WT-mCherry levels. Error bars indicate mean ± standard

Supplementary Figure 9.

(a)

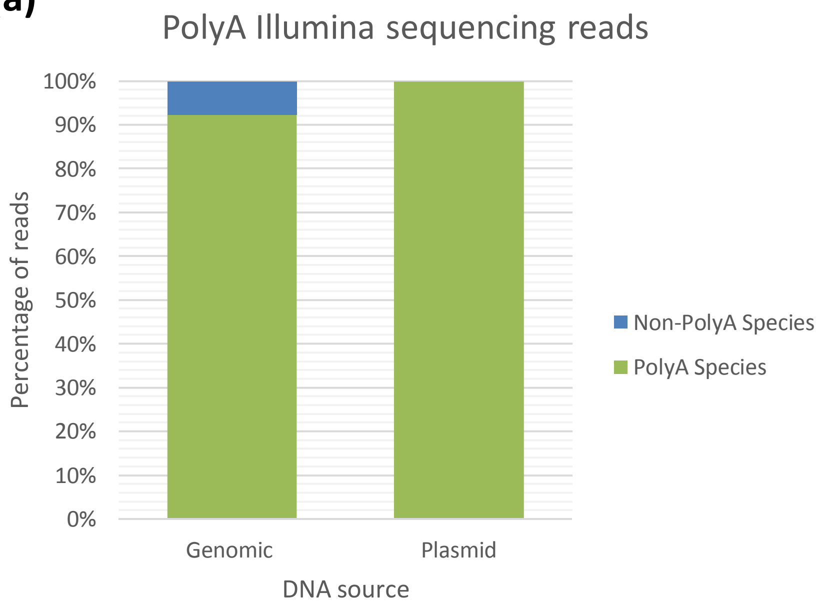

(b) Non-PolyA species of genomic reads (7.73%):

TGCACCCAAAAAAATTTACAAAAAAACCGTGAGCAAGGGCGA

Bold portion maps to chromosome X
Underline portion maps to chromosome 3L

Illumina sequencing of polyA track genomic insertion. Approximately 30 generations after insertion of HA-(AAA)12-mCherry, genomic DNA was sequenced to examine mutation rate of long PolyA tracks. The fraction of sequencing reads which contain a polyA track vs reads that do not are shown for both genomic DNA and plasmid DNA (a). The non-polyA species of reads from genomic DNA are shown (b).

Supplementary Figure 10

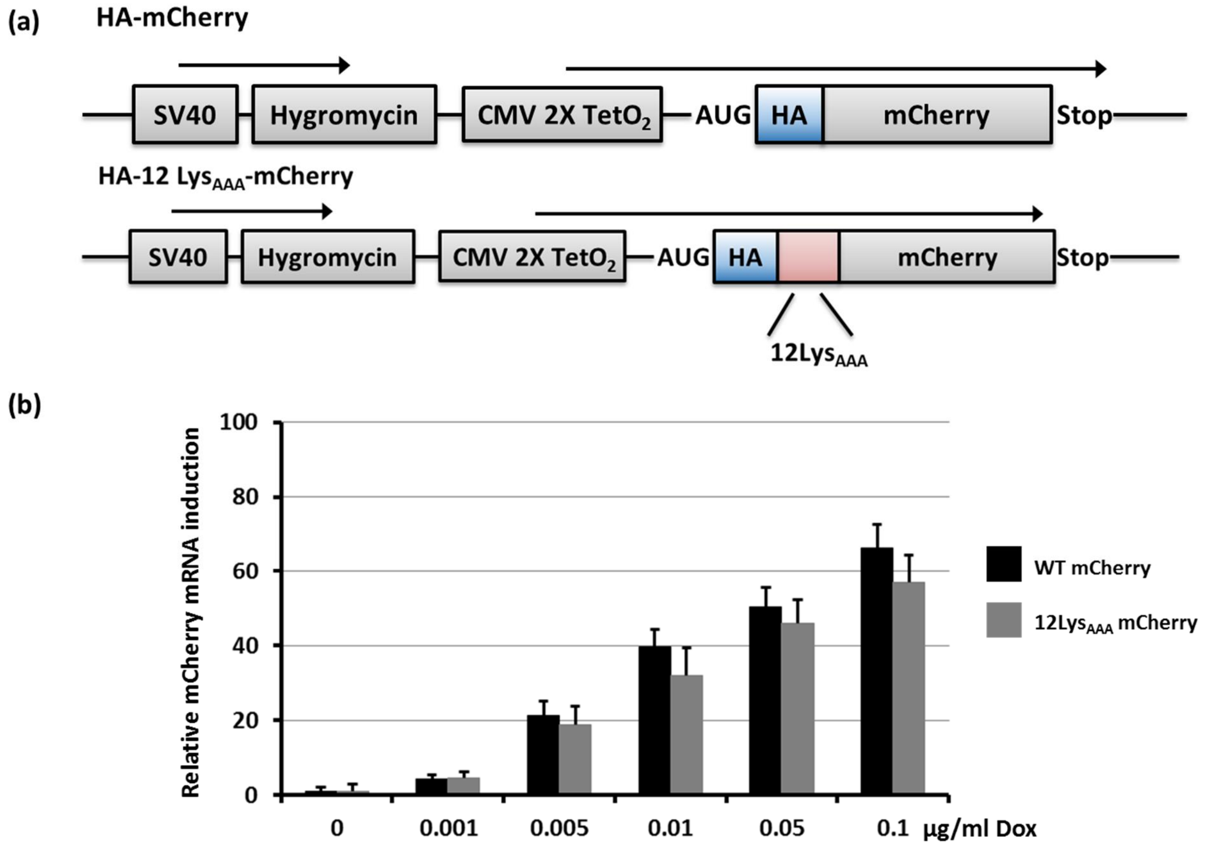

Diagram of mCherry expression constructs and their transcriptional activation in Flp-In^{TM} T-REx^{TM} 293 stable cell lines. (a) Scheme of genetic loci expressing WT (HA-mCherry) and 12Lys$_{AAA}$ insertion construct (HA-12Lys$_{AAA}$-mCherry) in stable Flp-In^{TM} T-Rex^{TM} 293 cell lines. Position of the SV40 promoter (SV40), hygromycin B phosphotransferase (Hygromycin), antibiotic resistance gene for selection of single insertion constructs), doxycyclin-inducible CMV promoter (CMV 2x $TetO_2$), double HA-tag (HA) and fluorescent reporter (mCherry) are indicated. Red box designates the position of in frame inserted 12 Lys$_{AAA}$ sequence. WT construct contains no insertions at this position. (b) Relative folds of transcriptional activation for WT and 12Lys$_{AAA}$ mCherry loci were calculated from mRNA levels for each construct at different levels of induction by doxycycline (Dox, 0.001-0.1µg/ml). RT-qPCR data for each construct was normalized to the mRNA levels of constitutively expressed hygromycin B phosphotransferase gene. Fold induction was calculated over the non-induced samples for each construct separately. Error bars indicate mean ± standard deviation.

Supplementary Figure 11.

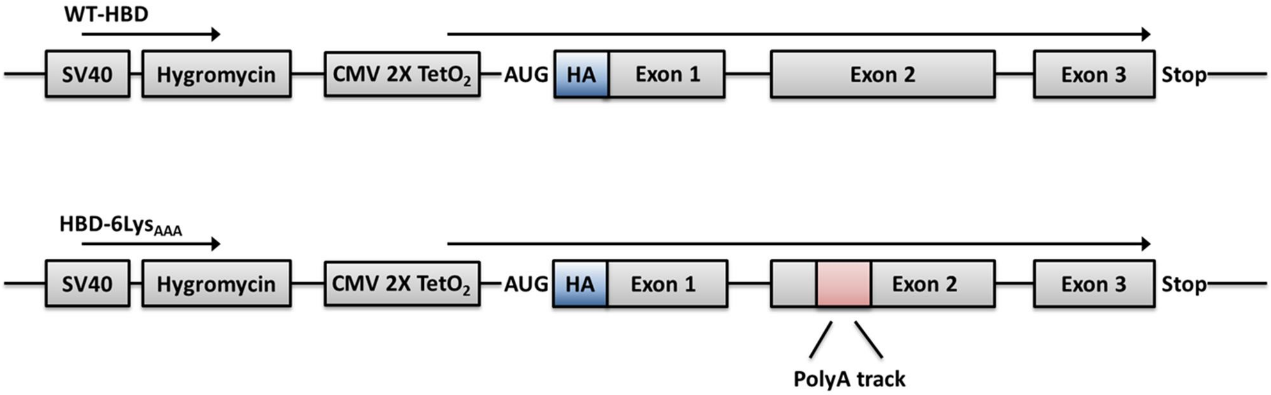

Diagram of human beta globin delta chain (HBD) expression constructs in Flp-In™ T-REx™ 293 stable cell lines. Scheme of genetic loci expressing WT-HBD and HBD-6Lys_{AAA} constructs in stable Flp-In™ T-REx™ 293 cell lines. Position of the SV40 promoter (SV40), hygromycin B phosphotransferase (Hygromycin), antibiotic resistance gene for selection of single insertion constructs), doxycyclin-inducible CMV promoter (CMV 2x $TetO_2$), double HA-tag (HA) and HBD reporter (mCherry) are indicated. Red box designates the position of in frame inserted 6 Lys_{AAA} sequence. WT construct contains no insertions at this position.

Supplementary Figure 12.

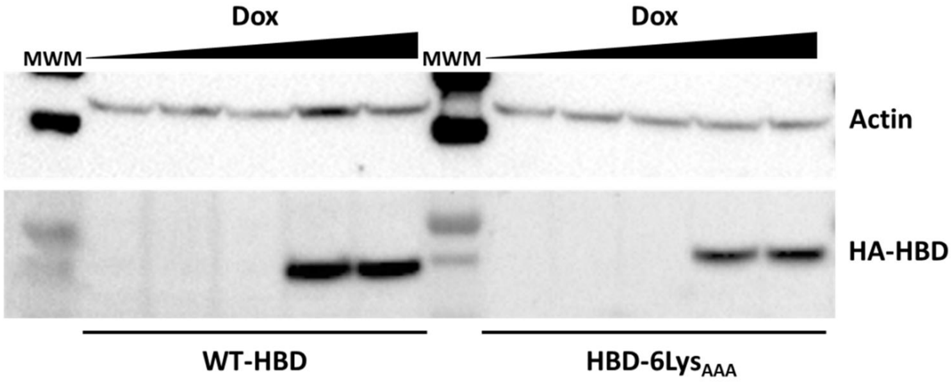

Western blot analysis of HBD protein abundance during Dox induction. Western blot analysis of the cell lysates from Flp-In™ T-REx™ 293 stable cell lines expressing doxycycline (Dox) inducible wild type (WT-HBD) and 6 Lys_{AAA} insertion construct (HBD-6Lys_{AAA}) from a single locus. Dox concentration in the media was varied from 0.0 to 1 μg/ml. Constitutively expressed β-actin (Actin) was used as a loading control and was detected using specific antibody. Positions of the HA-tagged HBD protein (HA-HBD), normalization control (β-actin) and molecular weight marker (MWM) are indicated.

Supplementary Figure 13.

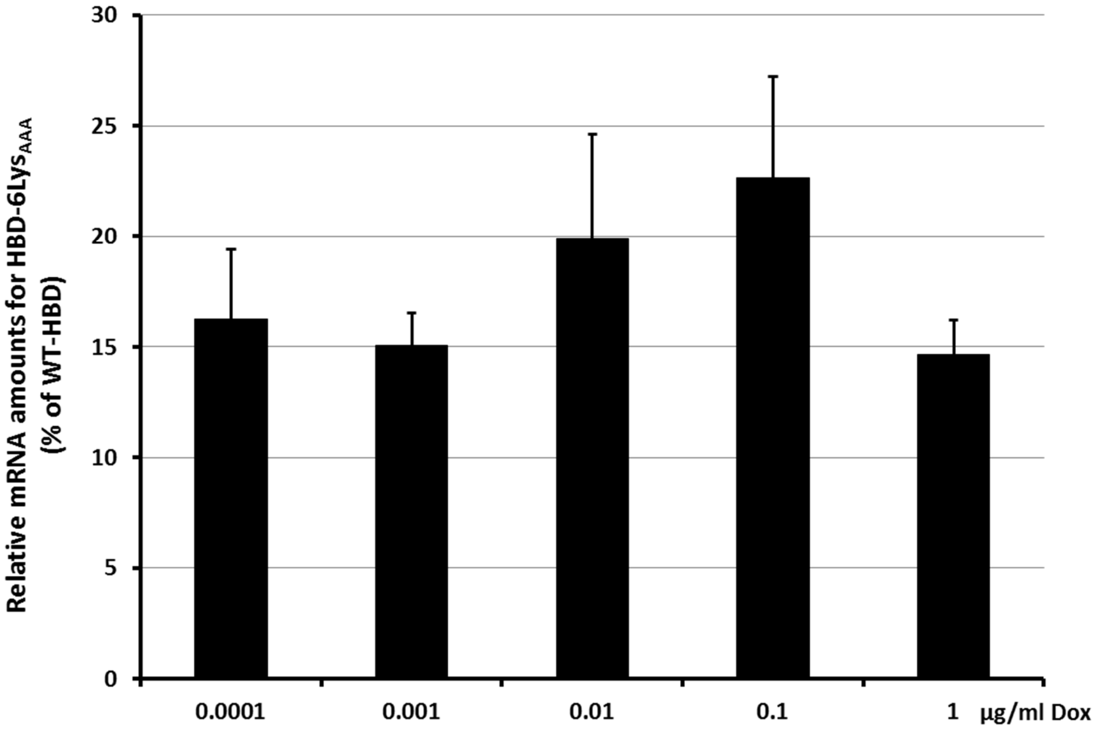

Ratio of WT-HBD and HBD-6Lys$_{AAA}$ mRNA abundance from Flp-In^{TM} T-REx^{TM} 293 stable cell lines. Steady state mRNA levels of the 6Lys$_{AAA}$ insertion construct (HBD-6Lys$_{AAA}$) measured by qRT-PCR. Relative levels of the mRNA for HBD-6Lys$_{AAA}$ are presented as percentage of the wild type HBD (WT-HBD) construct mRNA levels. Error bars represent mean± standard deviation values (n=3). Numbers indicate final concentration of Dox in the media.

Supplementary Figure 14.

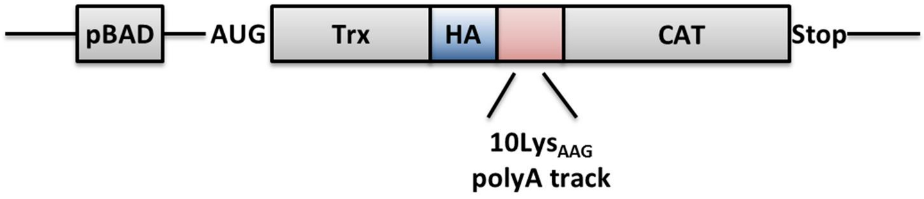

Diagram of chloramphenicol acetyltransferase (CAT) expression construct used in *E. coli*. Position of the inducible arabinose promoter (pBAD), Thioredoxin (Trx), double HA-tag (HA), insertion sequences (10 Lys$_{AAG}$ and polyA track (3-10Lys$_{AAA}$) and reporter gene (*CAT*) are indicated.

Supplementary Figure 15.

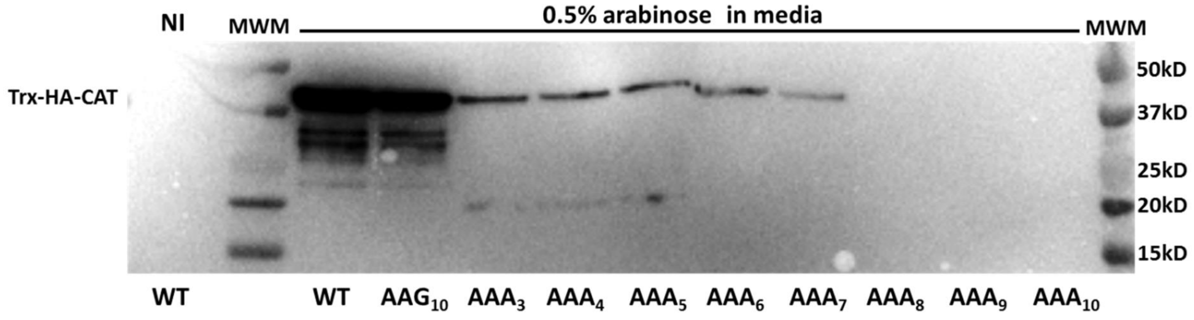

Expression of arabinose-inducible fusion Thioredoxin-HA-CAT constructs in E. coli. Western blot analysis of the lysates from *E. coli* cells expressing arabinose-inducible wild type Trx-HA-CAT (WT), 10 Lys_{AAG} (AAG_{10}) and 3-10 Lys_{AAA} insertion constructs (AAA_{3-10}). Cells were induced with 0.5% (w/v) of arabinose in the media for 30 minutes. Equal number of cells were harvested, lysed in SDS sample buffer and loaded on SDS-PAGE gel. Trx-HA-CAT fusion proteins were detected using HA-tag specific antibody. Positions of the Trx-HA-CAT proteins and molecular weight marker (MWM) are indicated. NI represents negative control; WT construct without induction.

Supplementary Figure 16.

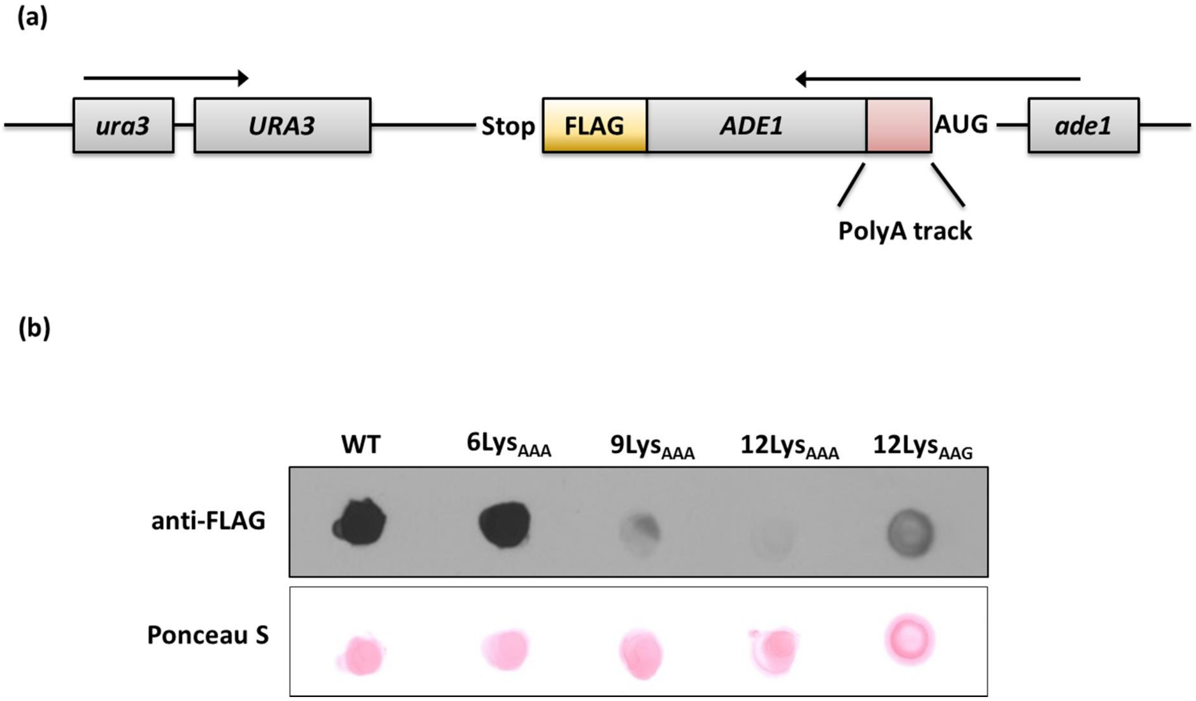

Diagram of N-succinyl-5-aminoimidazole-4-carboxamide ribotide synthetase (ADE1) construct and expression of ADE1 constructs in *S. cerevisiae*. (a) Position of the orotidine 5'-phosphate decarboxylase promoter and and gene(*ura3* and *URA3*, respectively), ADE1 promoter and gene (*ade1* and *ADE1*, respectively) and FLAG-tag (FLAG) are indicated. Red box (insert) designates the position of in frame inserted 12 Lys$_{AAG}$ and 6-12 Lys$_{AAA}$ sequences. WT construct contains no insertions at this position. (b) Dot blot of yeast cell lysates expressing FLAG-tagged WT, 12 Lys$_{AAG}$ and 6-12 Lys$_{AAA}$ ADE1 protein from endogenous *ade1* promoter. ADE1 protein was detected using anti-Flag (Sigma) antibody. 20 μg of total protein was spotted onto a nitrocellulose membrane for each construct. Ponceau S staining is used as loading control.

Supplementary Figure 17.

Image of the full western blot represented in the main Figure 2b. Degradation or frameshifted product TRX-HA is indicated. Orientation of the gel is as in the original figure.

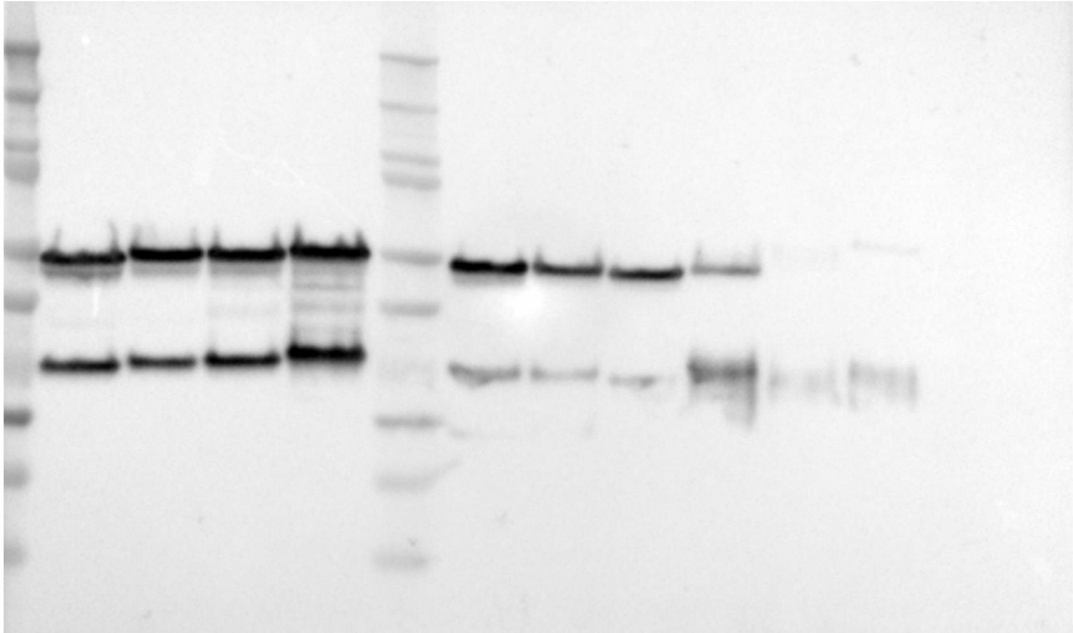

Supplementary Figure 18.

Eight biological replicas for experiment represented in the main Figure 3b. Loading of samples is in the same order as in the original figure for both mCherry and BAR (BASTA) western blots.

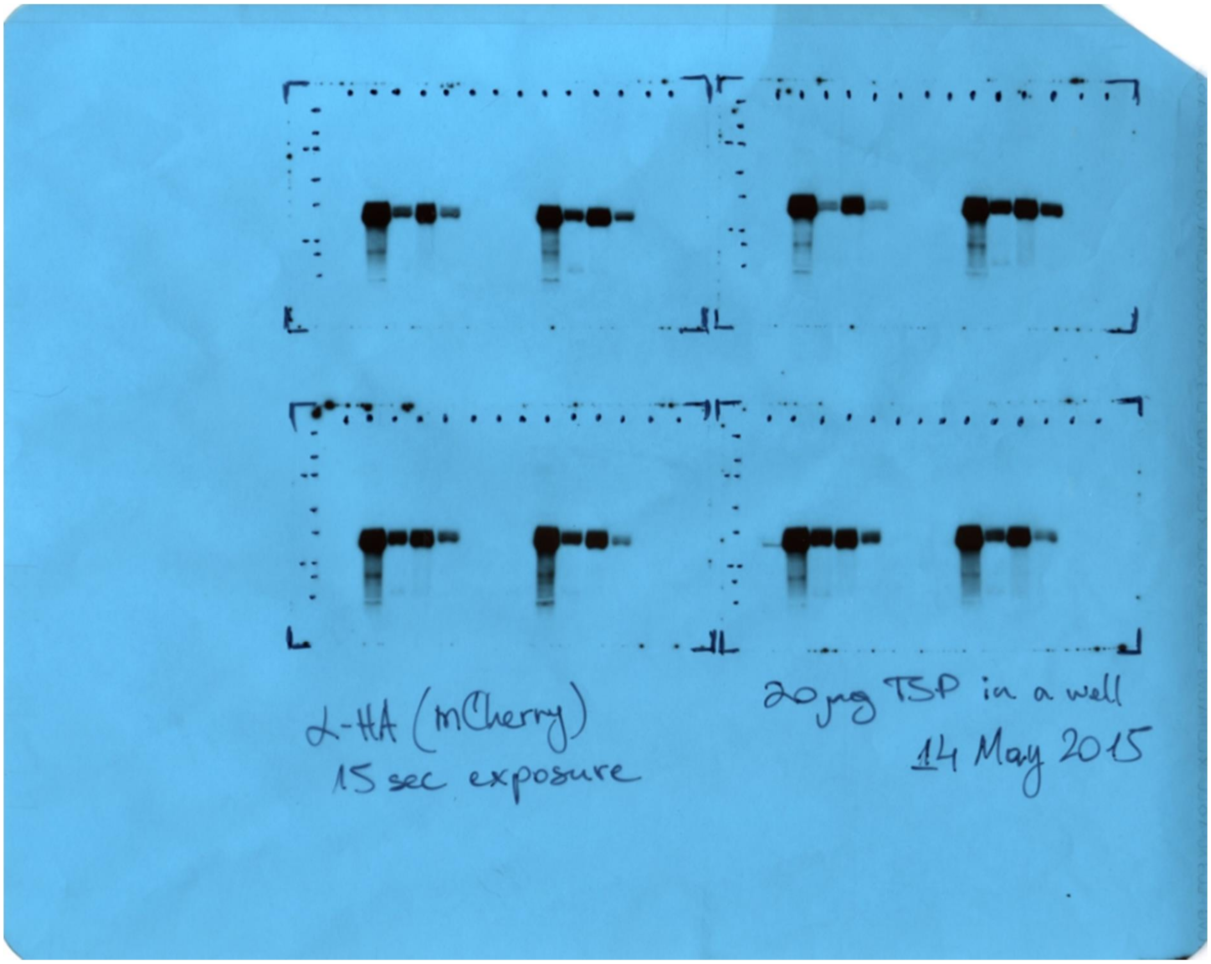

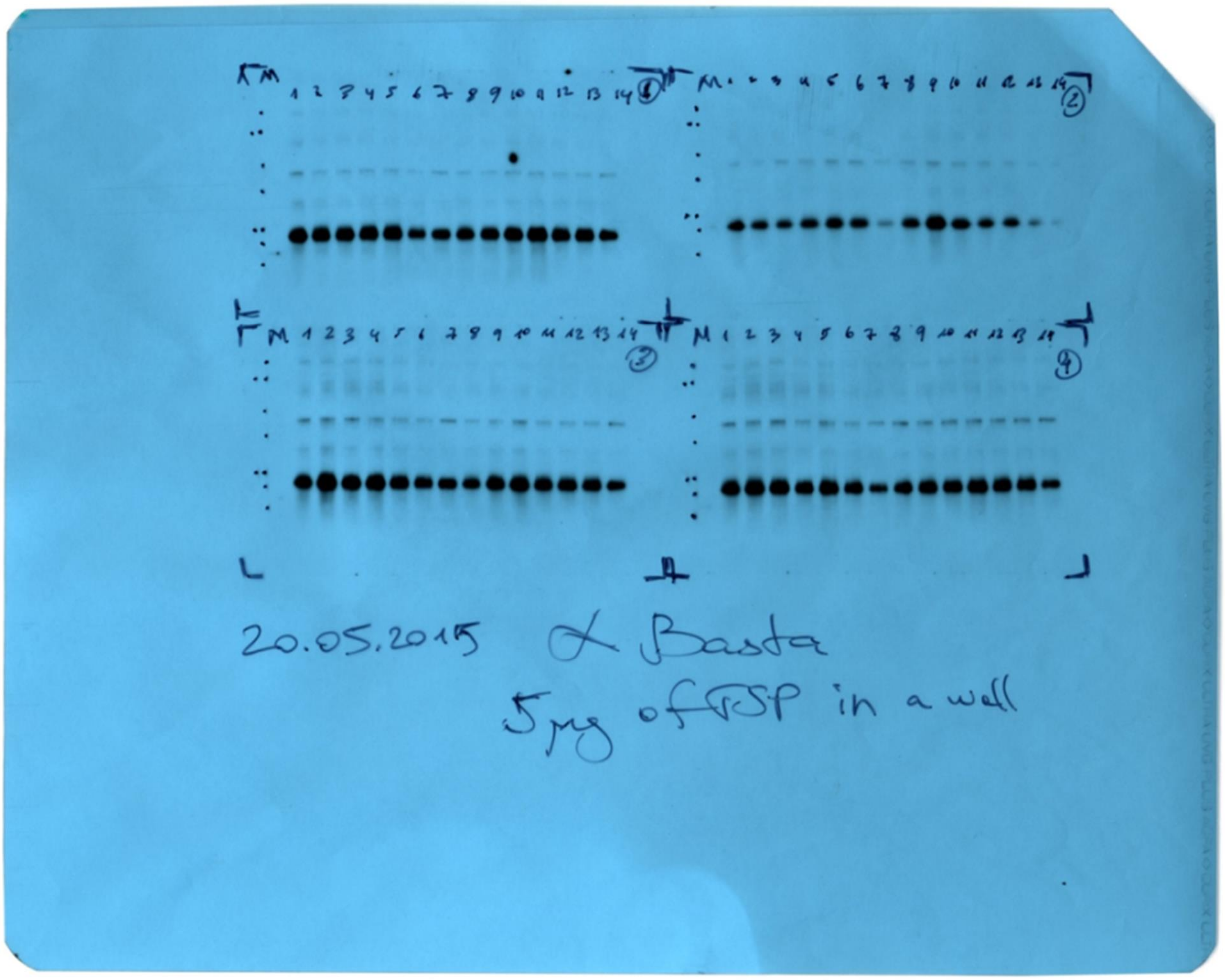
M 1 2 3 4 5 6 7 8 9 10 11 12 13 14 ①
M 1 2 3 4 5 6 7 8 9 10 11 12 13 14 ②
M 1 2 3 4 5 6 7 8 9 10 11 12 13 14 ③
M 1 2 3 4 5 6 7 8 9 10 11 12 13 14 ④
20.05.2015 α Basta
5 μg of TSP in a well

Supplementary Figure 19.

Complete image of the western blot used for the main Figure 3e. Loading and orientation of the western blot is the same as in the original figure. Samples from two biological replicas are shown on this image. Replica on the left side of the image is used for representation in the main figure.

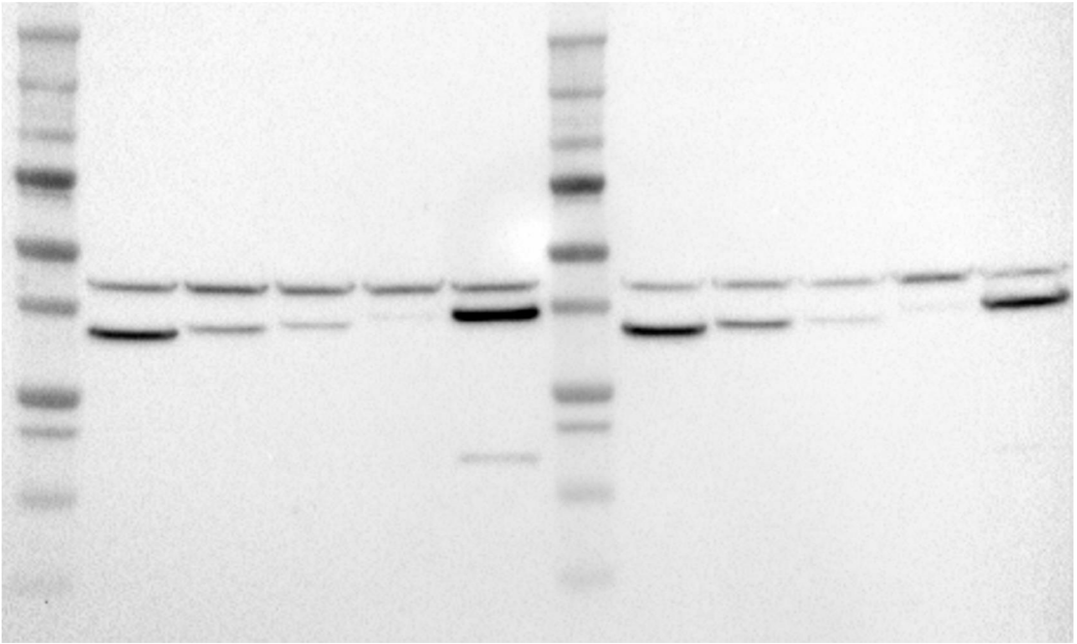

Supplementary Figure 20.

Image of the full western blot represented in the main Figure 5a. Low molecular weight band for degradation product (reacting with HA-antibody) and unspecific high molecular band (reacting with δ-tubulin antibody) are visible on the image. Orientation of the gel is the same as in the original figure.

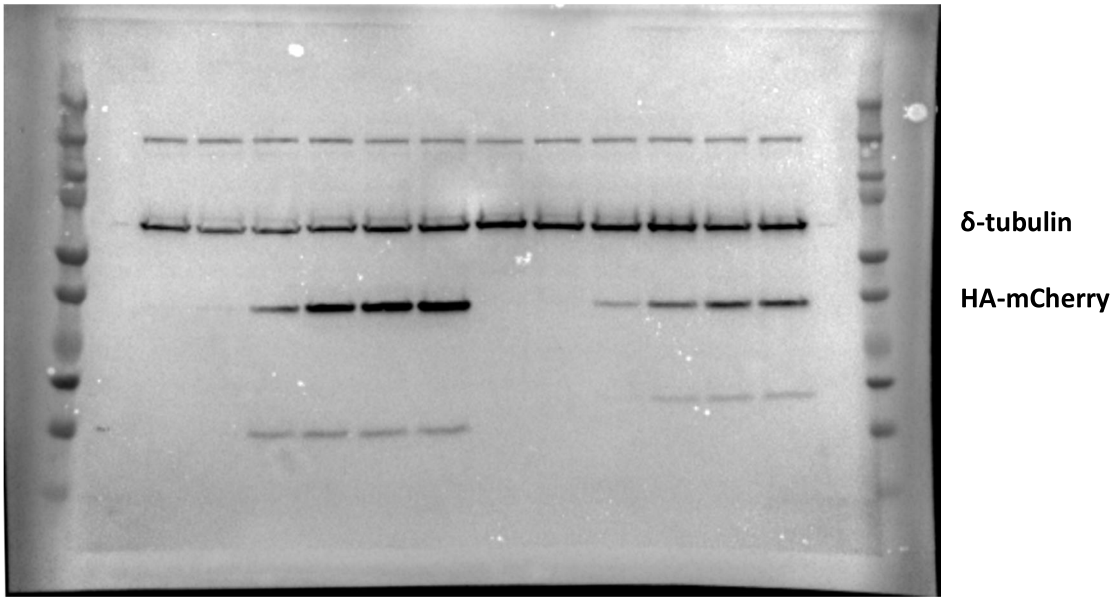

Supplementary Figure 21.

Original plates for the experiments described in Figure 6a. Two colonies for each construct are excised from the plates to make the final figure.

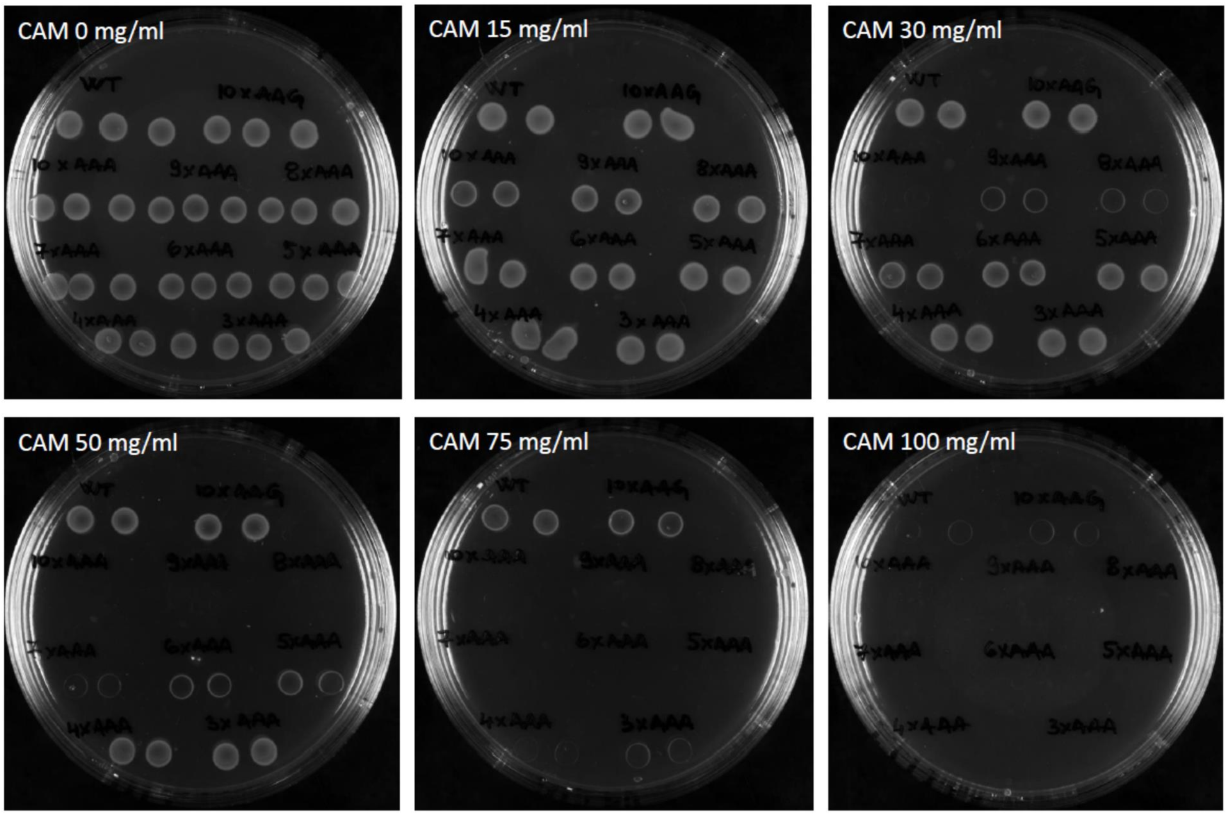

Supplement: Supplementary Information — Supplementary Figures [file ncomms14112-s1.pdf]
